# Impact of implementing a protocol of respiratory care measures and optimization of mechanical ventilation in potential lung donors

**DOI:** 10.5935/0103-507X.20200095

**Published:** 2020

**Authors:** Marco Guillermo Bezzi, Carla Candela Brovia, Juan Manuel Carballo, Maia Inés Elías, Agustina Belén Moreno, Vanesa Romina Ruiz, Fernanda Cordiviola, David Barbieri, Adriana Fariña, Silvina Borello

**Affiliations:** 1 Hospital General de Agudos D. F. Santojanni - Buenos Aires, Argentina.; 2 Instituto de Trasplante de la Ciudad de Buenos Aires - Buenos Aires, Argentina.; 3 Sección de Rehabilitación y Cuidados Respiratorios del Paciente Crítico, Hospital Italiano de Buenos Aires - Buenos Aires, Argentina.

**Keywords:** Lung transplantation, Organ transplantation, Donor selection, Tissue and organ procurement, Respiration, artificial, Brain death, Trasplante de pulmón, Trasplante de órganos, Selección de donante, Obtención de tejidos y órganos, Respiración artificial, Muerte encefálica

## Abstract

**Objective:**

To describe the results from the implementation of a respiratory care and mechanical ventilation protocol on potential lung donors who met the conditions for procurement. The secondary objective is to compare the results with historical data.

**Methods:**

This was a retrospective, observational study. It included potential donors suitable for procurement of organs who had brain death and were hospitalized in critical care units of the Autonomous City of Buenos Aires from April 2017 to March 2018. Main variables: number of potential lung donors that reached the objective of procurement, rate of lungs procured, and rate of implanted lungs. Values of p < 0.05 were considered significant.

**Results:**

Thirty potential lung donors were included, and 23 (88.5%; 95%CI 69.8 - 97.6) met the oxygenation objective. Twenty potential lung donors donated organs, of whom eight donated lungs, with which four double lung transplants and eight single lung transplants were performed. Seven of 12 lungs were procured and implanted in the preprotocol period, while all 12 were under the protocol (p = 0.38). The implantation rate was 58.3% (7/12) in the historical control period and 100% (12/12) (p = 0.04) in the study period.

## Abstract (es)

**Objetivo:**

Describir los resultados de la implementación de un protocolo de cuidados respiratorios y de ventilación mecánica en el potencial donante de pulmón, que cumplen las condiciones para ser procurados. El objetivo secundario es comparar los resultados con datos históricos.

**Métodos:**

Estudio retrospectivo y observacional. Incluye potenciales donantes aptos para procuración de órganos con muerte encefálica internados en las áreas críticas de la Ciudad Autónoma de Buenos Aires, desde abril de 2017 hasta marzo de 2018. Variables principales: número de potencial donante de pulmón que alcanzan el objetivo de procuración, tasa de pulmones procurados y tasa de pulmones implantados. Se consideraron valores significativos p < 0,05.

**Resultados:**

Se incluyeron 30 potenciales donantes de pulmón, 23 (88.5%; IC95% 69,8 - 97,6) cumplieron el objetivo de oxigenación. Veinte potenciales donantes de pulmón donaron órganos y de ellos, ocho donaron pulmones, con los cuales se realizaron 4 trasplantes bipulmonares y 8 unipulmonares. Los pulmones procurados e implantados en el periodo pre-protocolo fueron 7, mientras que durante el protocolo fueron 12 (valor p = 0,38). La tasa de implantación fue 58,3% (7/12) en el control histórico y 100% (12/12) (valor p = 0,04) en el periodo de estudio.

**Conclusión:**

El protocolo permitió alcanzar el objetivo de oxigenación en la mayoría de los potenciales donantes de pulmón y una mejoría estadísticamente significativa en la tasa de implantación.

## INTRODUCTION

Organ procurement and maintenance are hospital care activities that take place in the time elapsed from the diagnosis of brain death (BD) to its confirmation and the ablation of the organs, and their purpose is to provide the organs in a condition that will meet the health demand of the patients waiting for a transplant to treat their terminal, acute, or chronic diseases.^(1,2)^

In the organ and tissue procurement for transplantation, the lung is one of the organs that suffers the greatest impact in the context of BD, and its low availability worldwide is a consequence of multiple factors, including the processes associated with BD, fluid administration, and injury induced by forced mechanical ventilation (MV).^(2-4)^

The deterioration of gas exchange, evidenced by a fall in the arterial oxygen partial pressure/fractional inspired oxygen (PaO_2_/FiO_2_) index is one of the main reasons to stop considering the lungs for donation.^(5)^ In addition, the pulmonary lesions that occur in cases of BD due to traumatic mechanisms, the pulmonary lesions induced by MV, and the sensitivity of the lungs to infection (which is closely related to the time of invasive MV and the abolition of defense mechanisms) make the lung one of the first organs that is discarded in the procurement process.^(2,6,7)^

Although some studies suggest the application of a pulmonary protection strategy to improve the number and quality of the lungs procured, only one randomized clinical trial evaluated this strategy, and it was stopped prematurely.^(8-10)^

Current international guidelines for potential donors recommend a protective ventilatory strategy with low tidal volume (V_t_). However, there is still no strong evidence on the best ventilatory strategy after BD, and our country has no standardized protocol to maintain optimal ventilation.^(11-13)^

The objective of the present study is to describe the results of the implementation of protocolized management of respiratory care and MV in potential lung donors (PLD) by calculating the rate of lungs that met the conditions for procurement and implantation. The secondary objective is to compare the results with historical data.

## METHODS

A retrospective and observational study was carried out.

Consecutive PLD with BD who were suitable for organ procurement, were aged 18 to 65 years, were admitted to critical care units of institutions of the *Ciudad Autónoma de Buenos Aires* (CABA) between April 2017 and March 2018, were cared for by the *Instituto de Trasplante de la Ciudad Autónoma de Buenos Aires*, and met the criteria to be an ideal lung donor or had no more than one marginal lung donor criterion (Table 1) were included.^(14,15)^ Those with chronic lung disease, alterations in X-ray lung fields, evidence of bronchoaspiration, or the presence of purulent secretions with confirmed infection were excluded. Potential lung donors in whom the final data could not be collected due to protocol suspension and/or cardiac arrest were eliminated. A group of PLD with BD treated in the same period during the previous year was used as a control group.

**Table 1:** Acceptance criteria for lung donors

Variable	Ideal donor	Marginal donor
Age (years)	< 55	55 - 65
PaO_2_/FiO_2_ (mmHg) (PEEP 5cmH_2_O y FiO_2_ 1)	> 300	250 - 300
Time of mechanical ventilation (hours)	< 72	> 72
Smoking (packages/year)	< 20	> 20

PaO_2_/FiO_2_ - ratio between arterial oxygen partial pressure and fractional inspired oxygen; PEEP - positive end-expiratory pressure.

The main variables were:

- Rate of PLD that reach the procurement objective: expressed as the number of PLD with PaO_2_/FiO_2_ greater than 300mmHg at the end of the protocol, divided by the total number of PLD included.
- Procured lung rate: expressed as the number of procured lungs, either unilateral lung, bilateral lungs, or cardiopulmonary block, divided by the total number of donor organs.
- Rate of implanted lungs: expressed as the number of implanted lungs, either unilateral lung, bilateral lungs, or cardiopulmonary block, divided by the total number of lungs procured.

Secondary variables were: demographic characteristics of the PLD; ventilatory and gasometric parameters; duration time elapsed since the BD to organ ablation.

**Figure 1:**
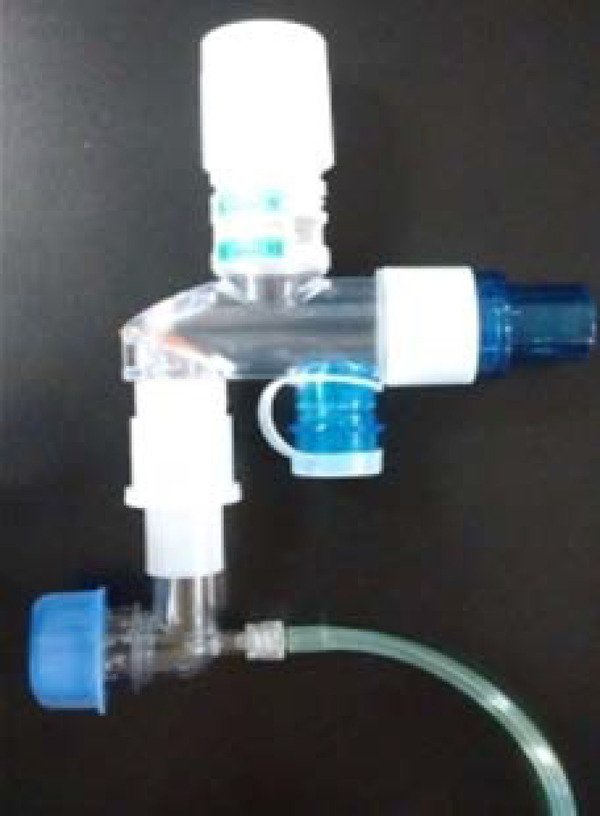
T piece with continuous positive airway pressure valve.

A protocol of MV and respiratory care measures was implemented in the PLDs to maintain adequate gas exchange, avoid MV-induced lung injury, and optimize the state of the organ to be procured. The schedule chosen to apply the ventilatory support was the volume-controlled continuous mandatory ventilation (VC-CMV) mode, with an initial V_t_ of 8mL/kg depending on the predicted body weight of each patient [(height in cm-152.4) × 0.91 + 45.5 in women or 50 in men]. The V_t_ was reduced to 5mL/kg during maintenance in the presence of plateau pressure ≥ 30cmH_2_O or insufflation pressure *(*driving pressure) ≥ 14cmH_2_O (Table 2).^(6,9,11,12,16)^ The initial positive end-expiratory pressure (PEEP) value was 5cmH_2_O and was modified according to the variations in FiO_2_ and the oxygenation objectives (PaO_2_ > 90mmHg or SpO_2_ > 95%) according to the table of the Acute Respiratory Distress Syndrome Network.^(17)^ When necessary and not routinely, aspiration of oropharyngeal and tracheobronchial secretions was performed. The criteria were as follows: sawtooth pattern in the flow-time curve of the ventilator monitor, auscultation of thick rales, increase in peak pressure, deterioration of oxygen saturation, visible airway secretions, and/or the need to obtain a sputum sample to rule out or identify pneumonia or other lung infections. A closed aspiration system was used.^(18,19)^ The usual measures of respiratory physiotherapy were adopted to avoid atelectasis, such as postural drainage with lateral decubitus. Airway humidification was performed with a heat and moisture exchanger. In the presence of hypothermia, active humidification was used. The apnea test was performed through a T-piece with a 10cmH_2_O PEEP valve and O_2_ flow of 10 - 12L/minute to minimize alveolar closure or collapse and thereby increase end-expiratory lung volume (Figure 1).^(6,20)^

**Table 2:** Protocol for mechanical ventilation and respiratory care

Mechanical ventilation	Respiratory care
Mode: VC-CMV	Apnea test: CPAP 10cmH_2_O, flow O_2_ 10 - 12 L/minute
V_t_: 6 to 8 (mL/kg of predicted body weight) RR: with PaCO_2_ target 35 - 45mmHg PEEP: > 5cmH_2_O	Bronchial hygiene therapy: CSS, positioning, insufflation, increased expiratory flow
FiO_2_: according to PEEP/FiO_2_ table Ti: 0.8 - 1.2 seconds	Humidification of airway: heat and moisture exchanger
I:E: > 1:2 Trigger: in order to avoid self-triggering Driving pressure: 7 - 14cmH_2_O	Prevention of respiratory infections: elevated headrest > 30°, endotracheal balloon pressure > 25cmH_2_O, oropharyngeal aspiration
Recruitment maneuver: when disconnections or drop in oxygenation	Avoid disconnections of the circuit

VC-CMV - volume-controlled continuous mandatory ventilation; CPAP - continuous positive airway pressure; V_t_ - tidal volume; RR: respiratory rate; PEEP - positive pressure at the end of expiration; CSS - Closed suction system ; FiO_2 _- fractional inspired oxygen; Ti - inspiratory time; I:E - inspiration and expiration ratio.

When faced with any eventual disconnection of the ventilatory support and with an oxygenation drop, a recruitment maneuver was applied if the patient had hemodynamic stability and no pneumothorax. The maneuver was interrupted in the presence of SpO_2_ < 88%, heart rate > 140 beats per minute (bpm) or < 40bpm, mean blood pressure < 60mmHg or a decrease greater than 20mmHg from the baseline value, and/or cardiac arrhythmia.^(21)^ Subsequently, ventilation continued according to the proposed protocol.

The present study was approved by the local institutional Ethics Committee (Approval No. 858, Research Ethics Committee *Hospital Santojanni*) and respected the considerations on the care of participants in clinical research expressed in the Declaration of Helsinki and the Guide for Research in Human Health (resolution 1480/11) of the Ministry of Health of the *República Argentina*. The study did not present any type of risk. All study data were treated with maximum confidentiality, anonymously, with access restricted to personnel authorized for the purposes of the study by the evaluating ethics committee in accordance with current legal regulations (National Law on Protection of Personal Data 25.326/00 (Law of Habeas data) and Law 26. 529/09).

### Statistical analysis

Continuous variables that assumed a normal distribution are reported as the mean and standard deviation (SD). Otherwise, the median and interquartile range (IQR) were used. The categorical variables are reported as presentation number and percentage, with their respective confidence intervals. To determine the distribution of the sample, the Shapiro-Wilk test was run. For comparisons between numerical variables, the t-test was run for paired samples, and otherwise the Wilcoxon test was used. For the comparison of rates between the period before implementation or control (April 2016 - March 2017) and during the implementation of the protocol (April 2017 - March 2018), the chi-squared test or Fisher's exact test was used, as appropriate. Values of p < 0.05 were considered significant. For the analysis, IBM Statistical Package for Social Science (SPSS) version 24.0 for Macintosh OS was used.

## RESULTS

In the study period, from April 1, 2017 to March 31, 2018, 30 lung procurement procedures were carried out. The clinical and demographic characteristics of the PLDs are shown in table 3.

**Table 3:** Demographic and clinical characteristics of potential donors

Potential donors	
Sex female	13 (43.3)
Mean age (years)	36.3 ± 11.5
Establishment	
Private clinic	20 (66.7)
Public hospital	7 (23.3)
Security forces hospital	3 (10)
Diagnosis	
Hemorrhagic stroke	16 (53.4)
Ischemic stroke	3 (10)
Severe TBI	4 (13.3)
FAI skull	4 (13.3)
Other	3 (10)
Days of MV at admission	1 [1 - 3]

TBI - traumatic brain injury; FAI - firearm injury; MV - mechanical ventilation. Results expressed as n (%), mean ± standard deviation or median [interquartile range].

**Figure 2:**
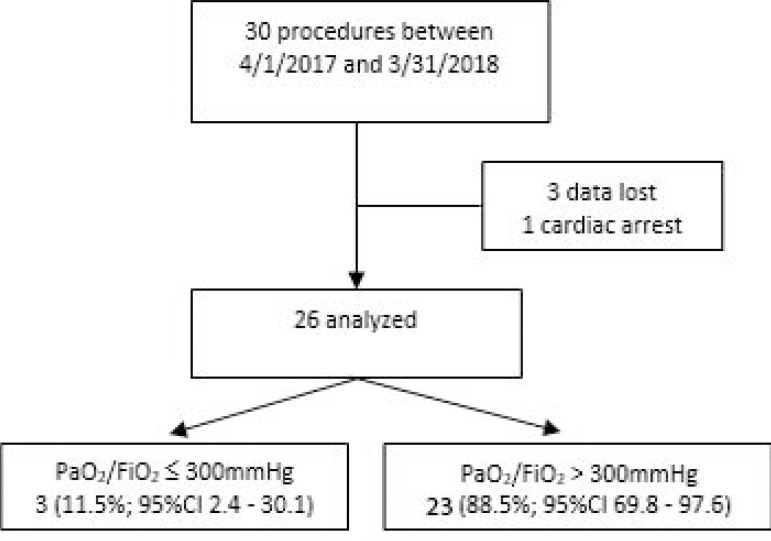
Flow chart. PaO_2_/FiO_2_ - ratio between arterial oxygen partial pressure and fractional inspired oxygen; 95%CI - 95% confidence interval.

In the main analysis, all 30 PLDs were included, four of whom were eliminated (in three, the data were lost, and one suffered cardiac arrest caused by refractory shock). The most frequent diagnosis of PLDs was hemorrhagic stroke. The median number of days of MV before the BD diagnosis was 1 day (IQR: 1 - 3). Sixty-six percent of the procedures were carried out in private clinics. Recruitment maneuvers (RM) were performed in 24 of the 30 PLD (80%) and had to be suspended five times due to hemodynamic instability. The median time elapsed from the diagnosis of BD to organ ablation was 12.5 hours (IQR: 6.9 - 16.6). Of the 26 PLD analyzed, 23 (88.5%) met the proposed oxygenation target, a PaO_2_/FiO_2_ greater than 300 at the end of the maintenance period (Figure 2).

In the analysis of the variables of ventilatory monitoring at the beginning of the protocol and at the end of the protocol (Table 4), no changes were observed in respiratory mechanics, ventilatory parameters, or blood gas analysis during the course of maintenance.

**Table 4:** Ventilatory parameters, respiratory mechanics, and blood gas analysis at the beginning and end of the protocol

Variable	Beginning	Final	p value
Vt (mL/kg)	7.8 [6 - 8]	7.4	0.65
Driving pressure (cmH_2_O)	8 [7.1 - 10.2]	9.5 [7.1 - 10.7]	0.1
Median plateau pressure (cmH_2_O)	15 [13 - 16.5]	15 [13 - 17]	0.25
PEEP (cmH_2_O)	6 (5 - 8)	6.83 (6.5 - 9)	0.62
Mean static compliance (cmH_2_O)	55 ± 13	53.5 ± 13.7	0.44
PaO_2_/FiO_2_	401 ± 98	397 ± 91	0.89

V_t_ - tidal volume; PEEP - positive end expiratory pressure; PaO_2_/FiO_2_ - relationship between arterial oxygen partial pressure and fractional inspired oxygen. Results expressed as median [interquartile range]; mean ± standard deviation.

**Figure 3:**
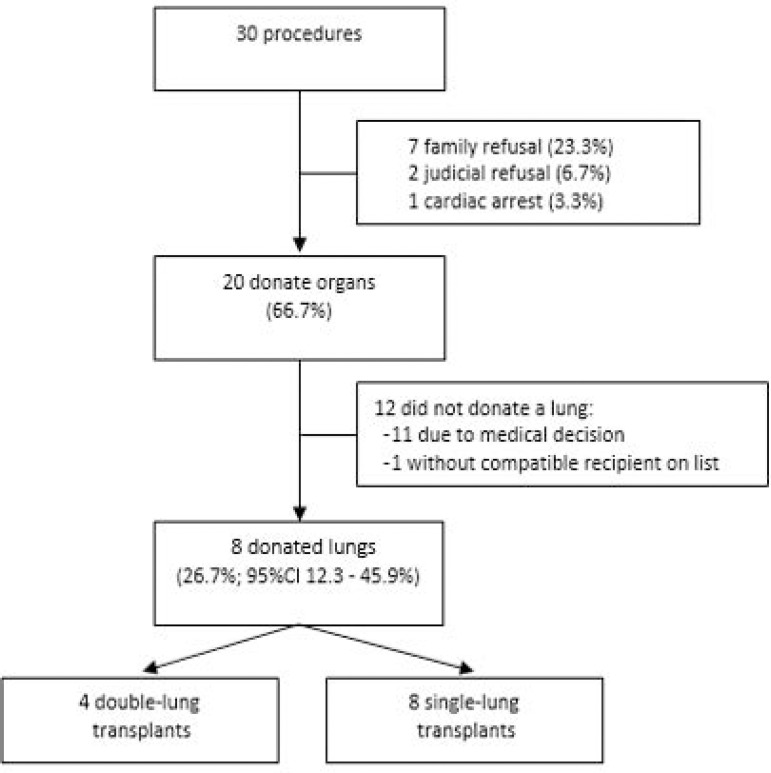
Description of the procedures. 95%CI - 95% confidence interval.

In ten PLD, the protocol was suspended. Of the 20 PLD treated, all donated organs, and 26.7% donated lungs, with which 12 patients were transplanted. In 12 PLD, lungs were not procured for various reasons, despite meeting the oxygenation criterion (Figure 3).

The procured and implanted lungs were compared to those from a historical control, the same period of the previous year (from April 1, 2016 to March 31, 2017). The rate of procured lungs in relation to total procured organs was similar in both periods, 12/220 (5.4%) in the control versus 12/229 (5.2%) in the study period (p = 0.91). The number of lungs procured and implanted in the control period was seven, while in the study period it was 12 (p = 0.38). The proportion of implanted lungs was 58.3% (95%CI 27.7 - 84.8%) in the historical control, while during the protocol it was 100% (95%CI 73.5 - 100%), with a p = 0.04 (Table 5).

**Table 5:** Lungs procured and implanted before and after implementation of the protocol

Variable	2016 - 2017	2017 - 2018	p value
	n = 229	n = 220	
Lungs procured and implanted	7	12	0.38
Proportion of implanted lungs	7/12 (58.3)	12/12 (100)	0.04

Results expressed as n or n (%).

Source: Central de Reportes y Estadísticas del Sistema Nacional de Información de Procuración y Trasplante de la República Argentina (Cresi-SINTRA). Reporte nacional de procuración y trasplante, período 2016-2017 y 2017-2018.

## DISCUSSION

The main finding of this study is that almost all of the PLDs after the implementation of the proposed protocol managed to reach the oxygenation objective at the end of the maintenance period. Although no significant difference was observed in the rate of procured lungs, all of these were implanted in the study period, compared to just over half in the historical control, indicating a significant impact in terms of avoiding the loss of procured lungs.

By applying a multimodal protocol, other authors have increased both the lungs eligible for donation (by oxygenation criteria) and their procurement rate.^(8,9,22,23)^ Even other authors used RM as an isolated strategy to improve gas exchange and the compliance of the respiratory system and counteract the effects of alveolar derecruitment that can occur after the apnea test because of the disconnection of the respirator or the fall of oxygenation.^(24-27)^ According to Miñambres et al.,^(8)^ RMs should be performed not only for lung deterioration but also routinely as a preventive strategy.^(8,9)^ Although RM are incorporated into numerous protocols, there is no strong evidence of their benefit, and there is still no consensus on what type of maneuver to perform. In our study, RM were considered to have rescued a drop in oxygenation or disconnection and were used in 24 PLD. However, they had to be interrupted five times due to hemodynamic deterioration during their application.

Oxygenation is one of the most influential variables in the acceptability of a lung for procurement. A PaO_2_/FiO_2_ ratio greater than 300mmHg is a criterion for donation, but in the study by Angel et al., a PaO_2_/FiO_2_ greater than 400mmHg was a decisive factor for organ acceptance.^(22)^ According to a report in Argentina, in the period 2009 - 2013, the average PaO_2_/FiO_2_ ratio of donors was 430mmHg.^(15)^ In the present study, the median PaO_2_/FiO_2_ at the end of the protocol was close to 400mmHg. However, the mean PaO_2_/FiO_2_ of the effective lung donors was 450mmHg, although the proposed oxygenation target was lower. These findings reflect that transplant teams prefer oxygenation levels close to 500mmHg. Therefore, the protocol should not only increase the oxygenation value but also maintain it during the procurement period to increase the organs eligible for transplantation.

The study has some limitations. First, due to its design, it does not demonstrate the superiority of the protocol applied over another strategy. Second, although marginal inclusion criteria were incorporated, the number of procedures during the year was small, similar to the previous year, which was used as a reference. Third, the decision to use the organs procured includes multiple circumstances related to the lungs, logistics, the recipient, and the preferences of the transplant team. Finally, the rate of implanted lungs was compared against a historical control, and we must note that some organizational and logistical circumstances may have changed between the periods studied. However, a similar length of time was considered-a whole year-to minimize any such effects.

On the other hand, our study has several strengths. We report the first such protocol based on evidence from international clinical practice carried out at the local level. We implemented a protective ventilation strategy (V_t_ 6 - 8mL/kg of predicted weight) due to its demonstrated efficacy in promoting the availability and eligibility of organs in potential donors and its clinical benefits in patients without pulmonary pathology. In addition, we monitored driving pressure (avoiding exceeding 14cmH_2_O) and plateau pressure (avoiding exceeding 30cmH_2_O) to adjust the V_t_ individually to optimize lung protection.^(6,9,12,16)^ Likewise, we highlight that during the time elapsed in each procedure, oxygenation levels stayed at acceptable values, demonstrating that the implementation of a respiratory care protocol could be beneficial for the maintenance of the lungs procured. Lastly, a consensus was reached among a multidisciplinary team composed of nurses, doctors specializing in organ and tissue procurement, thoracic surgeons, doctors specializing in intensive care, and respiratory kinesiologists, who all worked according to the nature of the patients waiting on the transplant list.

## CONCLUSION

The implementation of a protocol of respiratory care and mechanical ventilation in potential lung donors allowed almost all of the patients treated in the study period to reach the proposed oxygenation target at the time of ablation. In addition, all the lungs procured were implanted, a significant improvement over the previous period.
